# FMRI using balanced steady-state free precession (SSFP)

**DOI:** 10.1016/j.neuroimage.2011.10.040

**Published:** 2012-08-15

**Authors:** Karla L. Miller

**Affiliations:** FMRIB Centre, University of Oxford, Oxford, UK

**Keywords:** SSFP, FISP, Steady-state, FMRI, Functional, Brain

## Abstract

Steady-state free precession (SSFP) is a highly-efficient MRI pulse sequence that has been a fairly recent arrival in the functional MRI realm. Several methods for using balanced SSFP to detect the BOLD signal have been proposed to date and will be discussed in this review. After a brief introduction to the general properties of SSFP, this review describes the quite different approaches of transition-band and pass-band SSFP in terms of functional contrast mechanism. It then discusses the potential advantages of these techniques, followed by their challenges and shortcomings. Finally, it gives an overview of some applications considered to date and the author's perspective on where these techniques are headed. In the spirit of this special issue, the author also includes some of the personal history underlying her own explorations in this area.

## Pre-Ramble

**Fig. 1:**
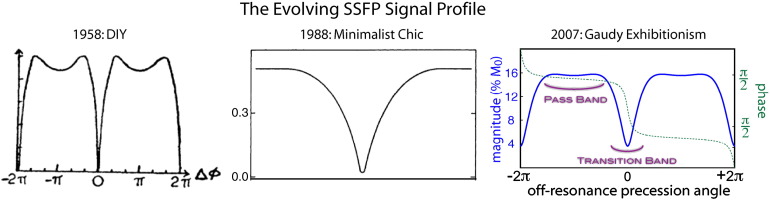
Evolution of the SSFP signal profile in the MR physics literature. The original 1958 version (Carr, 1958) boasted a distinct “Do-It-Yourself” feel, which had evolved by 1988 to a more minimalist form (Zur et al., 1988). By 2007, several major innovations had occurred, including a vast menagerie of fonts, gratuitous drop shadows and copious use of color (Miller et al., 2007). What a difference 50 years can make!

Steady-state free precession (SSFP, or more accurately, balanced SSFP^1^) is a magnetic resonance pulse sequence with a long history (Carr, 1958), despite being a relative latecomer to functional MRI. With the benefit of hindsight, one occasionally has the feeling that the 1958 NMR paper by Carr contains the “hard work” that underlies all subsequent SSFP-based methods. To those familiar with the technique, Carr's photographs of oscilloscope signal traces seem both quaint and prophetic, having been enthusiastically reproduced as the technique was re- and re-rediscovered in the context of MRI in the 1980s and 2000s (see Fig. 1). But to dismiss subsequent work as mere clever tweaks or serendipitous findings would downplay the acumen of those who have taken in the oddities and inconveniences of a notoriously complicated signal and seen novelty and opportunity. To be sure, SSFP has its objectively positive attributes: it can deliver the highest SNR efficiency of all known pulse sequences. But the enduring fascination it holds for many scientists, myself included, has more to do with its peculiar, occasionally troublesome, forever intriguing, properties. Recognizing that this sequence is less well known in the neuroimaging community, I will begin with a description of SSFP and its properties before moving on to its specific use in FMRI. The description of SSFP will necessarily be brief, and readers may wish to refer to: (Miller et al., 2011; Scheffler and Lehnhardt, 2003).

## A complicated signal

At first encounter, SSFP is a bit bewildering. The sequence itself is fundamentally very simple: a rapid train of identical excitation pulses applied every TR ms in the absence of gradients (or, more accurately, in the presence of “balanced” gradients that induce no net phase to the magnetization by the end of the TR). The key consequence is that the angle between the magnetization and the RF pulse depends on the precession induced by static field off-resonance during the TR, which is kept very short (TR = 2–20 ms). This is in distinction to most imaging sequences, in which the gradient waveforms are designed to induce particular precession angles during the TR (for example, the use of “spoiler” gradients to induce a range of precession angles across a voxel). As a result, the effect of the RF pulse on the magnetization depends on its resonance frequency. For the first few seconds of rapid RF pulsing, the signal oscillates wildly, then eventually settles into a frequency-dependent steady state with a characteristic “SSFP frequency profile” (Fig. 1). The shape of the profile, and the signal more generally, is dominated by resonance frequency and flip angle rather T1, T2 or other properties that more commonly dictate contrast in MRI (although it is often pointed out that the signal has T2/T1 contrast under common conditions (Zur et al., 1988)). These properties are quite unique and remarkable given that the sequence could hardly be simpler.

In general, the SSFP profile can be divided into “pass-band” regions, which are relatively insensitive to resonance frequency, and “transition-band” areas of high frequency sensitivity. The FMRI methods presented here are categorized as either pass-band or transition-band techniques, so it is useful to describe some basic properties of the SSFP profile. First, since the angle ϕ between the RF pulse and the magnetization drives signal behavior, the magnitude profile is periodic, repeating every TR^− 1^ Hz (ϕ is the same for all frequencies with ϕ + 2πn precession per TR). This period dictates that the bands become broader in absolute frequency with shorter TR. The profile itself is not fixed relative to absolute resonance frequency, but can be shifted along the frequency axis by linearly incrementing the RF pulse phase from one TR to the next (so, for example, an RF pulse that increments 15° each TR will perfectly track magnetization that precesses 15° in that time, and it has the same signal as on-resonance magnetization when no increment is used). Finally, the signal phase varies non-linearly with frequency (green dashed line in Fig. 1), with roughly constant phase in the pass band and an abrupt phase shift of 180° in the transition band (note that adjacent periods of the magnitude profile differ by 180°, such that the phase profile repeats every 2 TR^− 1^ Hz).

**Fig. 2:**
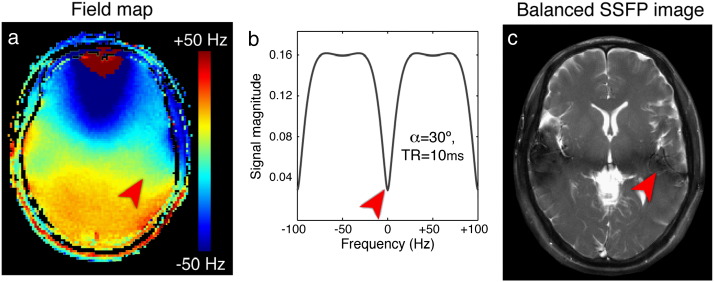
The frequency dependence of the SSFP signal magnitude results in spatial variation in the signal across the brain when the field is not homogeneous. Here, the field-map image on the left translates into the SSFP image on the right (where TR = 10 ms, so the transition-bands occur every 100 Hz). The red arrows indicate on-resonance (0 Hz) in all three subfigures, leading to a dark “band” in the SSFP image.

Most applications of SSFP focus on the pass-band region, which can achieve high SNR per unit time while being relatively insensitive to the precise off-resonance frequency. Provided the anatomy of interest can be shimmed such that the range of resonance frequencies is “contained” within the pass-band (typically about 0.75 × TR^− 1^ Hz), the image will have fairly uniform signal and contrast. If this level of field homogeneity cannot be achieved, the image will contain signal variations in anatomical regions that lie in the transition band (most typically, low signal “banding”, see Fig. 2). Pass-band SSFP was first explored for imaging in the 1980s when the gradient performance available at the time required TR ≥ 20 ms. These limits resulted in a narrow pass band with prohibitive shim requirements (about 40 Hz), and SSFP was deemed interesting but impractical. It was not until the late 1990s that gradient hardware was able to achieve TR = 2–5 ms, reducing the sensitivity to field inhomogeneity (so the pass band was 200–500 Hz wide), and SSFP suddenly became a real possibility for imaging.

As pass-band SSFP became increasingly viable as an imaging technique, the unique properties of the SSFP profile became a topic of interest. In the early 2000s, there was an explosion of innovative SSFP methods as several groups began to experiment with sculpting the profile into different shapes or using the frequency sensitivity of the transition band as a source of contrast.^2^ Without sacrificing the elegant simplicity of the SSFP sequence, these methods were able to achieve image contrast in unconventional ways. The use of SSFP for FMRI is one example of the broad range of pass-band and transition-band techniques that came from this innovative period.

## A novel idea

**Fig. 3:**
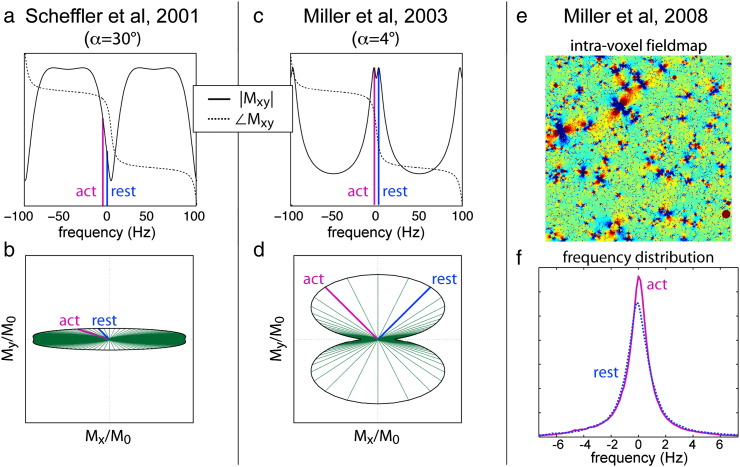
Transition-band SSFP FMRI uses the frequency-sensitive part of the profile to detect the frequency shift associated with BOLD activation (assuming TE/TR = 5/10 ms). In (a–d) a frequency shift of Δf = 5 Hz is demonstrated for active (magenta) and resting (blue) states. In Scheffler's method, the voxel lies slightly to one side of the central frequency, and Δf introduces a change in signal magnitude (a). In this picture, a relatively high flip angle is used. Our group proposed an alternative method based on the signal phase, which causes the signal to spread out unevenly in the transverse plane: in (b,d) the radiating lines indicate Mxy with 1 Hz spacing. If the resting and active frequencies straddle the center frequency, a 5 Hz frequency difference can induce a 90° phase shift (d). This allows a large signal change in the highest-signal portion of the SSFP profile (note a–d are all scaled the same). Most functional imaging aims to achieve contrast in inhomogeneous tissue, which has a complicated spatial variation in field (e). In this case, signal changes are not a simple frequency shift, but rather a change in the distribution of signal (f).

In 2001, Klaus Scheffler proposed a method for using the frequency sensitivity of SSFP to detect the frequency shift associated with the BOLD response (Scheffler et al., 2001).^3^ To explain the concept, it is helpful to start with the simplest case: a voxel entirely contained in a blood vessel, with resonance frequency proportional to the blood oxygenation. For example, in a vein parallel to B_0_ in which oxygenation changes from 70 to 85%, the frequency will shift by about 5 Hz (assuming B_0_ = 3 T). If our vessel lies in the transition-band region of the SSFP profile, the signal magnitude will fluctuate due to this relatively small frequency shift (see Fig. 3a). Further, the signal changes can be quite large due to the signal differential across the transition band. Scheffler reported 9–10% signal changes during a visual stimulus compared to a 3% change in gradient echo (GRE). This method provides a means of detecting the BOLD frequency shift directly, with the slight complication that the same frequency shift can create a positive or negative signal change, depending on which side of the transition band the voxel occupies. Of course, the BOLD frequency shift in a blood vessel can also be detected with a conventional GRE sequence as a change in signal phase; the key difference lies in the timescale (TE) over which this is achieved, a point to which I will return.

Scheffler's method represented a truly novel approach to achieving BOLD contrast. One disadvantage of the technique as published, however, was that functional contrast was tied to the part of the profile with lowest signal (the nulls in Fig. 3a). In 2003, our group proposed a variation on this technique^4^ that avoids this problem by taking advantage of two fairly obscure properties of the SSFP frequency profile (Miller et al., 2003). First, the magnetization undergoes an abrupt 180° phase shift over the transition band (dashed lines in Figs. 3a,c). Naturally, the magnetization phase evolves during the TR (see Fig. 9d in (Miller et al., 2003)), and the profile is here depicted at TE = TR/2, which reflects the average phase during the TR. Second, reducing the flip angle causes the magnitude profile to “invert”, with high signal in the transition band and low signal in the pass band (solid line in Fig. 3c). The combination of these features provides sensitivity to BOLD frequency shifts based on the phase while imaging in the high-signal portion of the profile. In the case of our vein with a 5 Hz shift, the phase would change by 90° in the region of high signal, producing about 4 times greater signal difference than can be obtained at high flip angle (compare Figs. 3b and d). This suggests that at low flip angle we would observe a large phase change in our blood vessel, which we did indeed observe in the sagittal sinus.

For either method, the picture of a voxel characterized by a single frequency is overly simplistic. The more realistic scenario would be an inhomogeneous voxel containing capillaries and extravascular tissue. In this case, the voxel contains a distribution of frequencies (Figs. 3e,f), and the voxel magnetization is fanned out across the profile in Figs. 3b,d. The total signal is the sum of the signal at each frequency weighted by its distribution fraction (i.e., a partial volume effect). Activation narrows the frequency distribution, causing the magnetization to cluster more coherently in the transverse plane, and thereby altering the total signal magnitude. This model, initially sketched in Scheffler's original paper and later explored in detail by myself (Miller and Jezzard, 2008), explains why low flip-angle SSFP results in signal magnitude rather than phase changes in gray matter. At higher resolution there is evidence for both magnitude and phase changes, presumably reflecting a range of partial volume effects in different voxels (Lee et al., 2007).

The picture of transition-band SSFP spreading the magnetization out in the transverse plane is reminiscent of the dephasing that occurs in GRE-based BOLD imaging. In GRE imaging, the voxel magnetization accrues phase spread linearly in time and without any dependence on the center frequency of the voxel. By comparison, in transition-band SSFP, the pass band has little or no phase spread while the transition band exhibits greatly amplified dephasing. Moreover, this transition-band sensitivity to frequency is intrinsic to the steady state, even at very short TE. For example, a 90° phase difference across a 5 Hz frequency separation would take TE = 50 ms to accrue with a GRE sequence, but is independent of flip angle, TE and TR in SSFP (although it does depend on T2). One direct consequence of this de-coupling of BOLD contrast from TE is flexibility in k-space acquisition. While GRE BOLD typically uses single-shot EPI (or spirals), SSFP is compatible with short, low-distortion readouts of a few milliseconds duration acquired over several shots. Moreover, these readouts are achieved without sacrificing time efficiency since no “dead time” is required to wait for contrast to accumulate.

## A different approach

Pass band SSFP FMRI appears to have been developed independently by several groups. The first discussion of BOLD effects in pass-band SSFP that I am aware of appeared in the cardiac MRI literature, where SSFP was already being touted for its excellent contrast between blood and myocardium. A few cardiac groups recognized that the T2/T1 contrast in SSFP could be used to detect BOLD T2 changes, initially for myocardial (Wright et al., 2001) or angiographic (Brittain et al., 2003) contrast.

Rohan Dharmakumar took up this topic in earnest during his PhD work with Graham Wright,^5^ with the goal of quantifying blood oxygenation (Dharmakumar et al., 2005). While they were aware of Scheffler's work, the strict shim requirements of transition-band SSFP were deemed incompatible with quantification and they focused instead on the pass band. Dharmakumar provided the first rigorous theoretical treatment of BOLD effects in SSFP by extending the Luz–Meiboom model (although this work was confined to the center of the pass band). This model accounts for the interaction between RF pulses and the exchange of water between red blood cells and plasma; depending on whether RF pulses are fast or slow relative to exchange times, these compartments may appear separate or mixed. In general, the compartments will be partly mixed, and the observed T2 will depend on the pulse interval as well as oxygenation. Dharmakumar's insight was to recognize that the excitation pulses in an SSFP experiment play a similar role to the refocusing pulses in the multi-spin-echo sequences considered in the classic Luz–Meiboom model.

The first demonstration of FMRI activation in the pass band was reported by Chris Bowen at the 2005 ISMRM (Bowen et al., 2005). This work followed close on the heels of Dharmakumar's early conference presentations, but was developed independently.^6^ At that time, Bowen was using SSFP to detect iron oxide particles, which are characterized by sub-voxel field perturbations similar to those shown in Fig. 3e. It was from this background that he tried a pass-band SSFP FMRI experiment, with the idea that the majority of the voxel might lie in the pass band, but the dipolar fields surrounding blood vessels would lead to microscopic banding. While later work points to other sources as the primary contributor of contrast, Bowen's results included a compelling comparison between SSFP and GRE scans that differed only by a spoiler gradient, showing that SSFP could detect activation at short TE while GRE could not.

The source of pass-band BOLD contrast would prove to be a topic of interest at the next ISMRM, when three groups proposed different contrast mechanisms: Dharmakumar's T2 contrast (Lee et al., 2006b),^7^ diffusion in dipolar field patterns (Bowen et al., 2006) and conventional T2* (Zhong et al., 2006). It would eventually become clear that all of these contrast mechanisms contribute to signal changes, with the primary mechanism depending on the protocol (particularly the TR and flip angle) (Miller and Jezzard, 2008).^8^ At short TR, the primary source of signal change in the pass-band is due to the change in apparent T2 as spins diffuse in the field patterns surrounding the microvasculature (Bieri and Scheffler, 2007; Dharmakumar et al., 2006) (Fig. 3e). At long TR, SSFP exhibits T2* dephasing, and the sequence behaves essentially like a poorly-spoiled GRE (Zhong et al., 2007). There may also be a contribution from the frequency shift itself (i.e., a transition-band effect away from the center of the pass-band) (Miller and Jezzard, 2008), although most studies aim to minimize this by choosing a flip angle with a maximally flat pass band.

The principal attraction of imaging in the pass band is less stringent shim requirements: where the “sweet spot” of the transition band occupies only about 10–15% of the frequency axis, about 75% of the frequency range is in the pass band. This technique is therefore much more compatible with whole-brain coverage than transition-band SSFP, particularly if very short TR can be achieved to broaden the pass band. The downside is reduced functional contrast: pass-band SSFP has smaller signal changes than can be achieved with conventional GRE at long TE (Miller et al., 2007; Zhong et al., 2007), and much smaller changes than transition band SSFP.

## The downside of frequency sensitivity

As discussed above, the primary advantage of transition-band SSFP for FMRI is the ability to amplify small frequency shifts into large signal changes. Typical transition-band signal changes are about 10–20%, although we have observed signal changes as large as 40% (Miller et al., 2006). From this perspective, it may seem surprising that pass-band SSFP has been the more popular technique in recent years, having lower contrast than both transition-band SSFP and conventional GRE BOLD. The primary disadvantage of transition-band SSFP stems from the very same frequency sensitivity that provides such strong functional contrast.

In both transition- and pass-band SSFP, the presence of bands due to frequency inhomogeneity makes whole-brain coverage difficult to achieve. This is particularly a problem in transition-band SSFP, since functional contrast is achieved over a very narrow range of frequencies. Tuning the RF phase cycling increment enables the user to shift the bands and thereby control where in the brain functional contrast is achieved. One of the first modifications to the transition-band technique was to combine activation maps acquired with several phase cycling increments to increase coverage (Miller et al., 2003), an established trick in the SSFP community (Bangerter et al., 2004; Vasanawala et al., 2000). A number of schemes for combining across different increments have since been proposed (Lee et al., 2008; Miller et al., 2006; Park et al., 2011). This solution is not particularly satisfying, however, as the entire experiment has to be repeated with a different phase cycling increment, reducing efficiency and incurring awkward experimental design requirements. More recently, methods for obtaining whole-brain contrast in a single scan have been suggested through either re-shaping the SSFP profile (Bieri et al., 2009) or rapidly transitioning between phase cycling increments (Patterson et al., 2011).

In pass-band SSFP, the requirements for field inhomogeneity are greatly reduced since the pass band spans much more than 50% of the frequency axis. Thus, even if the field homogeneity is insufficient to encapsulate the entire brain, two phase-cycling increments are sufficient to achieve whole-brain coverage (Lee et al., 2008). Depending on the achievable TR and quality of shim, pass-band SSFP may be able to contain most of the brain within a single pass band. One elegant approach to maximizing the coverage in a single pass-band scan is to modify shimming protocols to the specific problem at hand: rather than maximizing the overall frequency homogeneity, one can aim to maximize the *signal* homogeneity by penalizing only frequencies that cause the signal to lie in the transition band (Lee et al., 2009).

A related issue in transition-band SSFP is time-varying frequency, including both slow drift in the main magnetic field (e.g., due to gradient-induced heating) and faster drift from physiological sources (e.g., respiration). These drifts cause the profile to shift across the brain during the imaging experiment, and can be severe enough that a voxel that is in the transition band at the beginning of an experiment is in the pass band later in the experiment. Unlike physiological noise artifacts in conventional GRE BOLD, this is a multiplicative rather than additive effect (i.e., the drift modulates the strength of contrast in a voxel) and cannot simply be regressed out of the data.^9^ One solution that has been proposed is to measure the drift in real-time and adapt the phase cycling scheme to track the drift (Lee et al., 2006a; Wu et al., 2007). Thus, the frequency may drift over time but the bands will be spatially fixed. Provided the drift is relatively slow and global, this can be achieved without disturbing the steady state.

Non-balanced SSFP is one alternative for short TR imaging that avoids the inconveniences imposed by banding patterns (Barth et al., 2010). We have neglected these sequences in the present review in part because it is not yet clear how this new approach will fit into the pantheon of FMRI methods, but also because they do not share many of the properties of balanced SSFP discussed above. Nevertheless, it will be interesting to see these methods mature in future years, particularly at high field strengths.

## 2D or not 2D?

The primary draw of SSFP techniques is the potential to uncouple contrast from the image artifacts associated with conventional (GRE-EPI) BOLD FMRI: namely, distortion and dropout, both a direct result of field inhomogeneity. Dropout can be partially mitigated in GRE-EPI through improved shimming, slice orientation or reduced voxel size, but a method that could avoid dropout without sacrificing contrast would be enormously helpful. In SSFP, dropout is traded off for banding artifacts, which can be controlled (but not eliminated) through short TR and phase cycling, as discussed above. Distortion (blurring in the case of spirals) results from the use of long, single-shot readouts, and is one limitation on spatial resolution in GRE BOLD. Although multi-shot readouts are possible with GRE, there is a fundamental tradeoff: the more the k-space acquisition is segmented over multiple TRs, the less efficient the acquisition becomes due to the need for long TE to achieve functional contrast. This tradeoff is considerably less severe in SSFP since contrast does not require long TE, and high SNR can be achieved at short TR. The primary constraint is that SSFP is fundamentally a 3D k-space technique, which carries both advantages and disadvantages.

In SSFP, the magnetization can only be maintained in a steady state through relatively short TR, which essentially precludes 2D multi-slice imaging. For proof-of-principle, early SSFP FMRI acquired a single slice with single-line (2DFT) k-space acquisitions (Bowen et al., 2005; Miller et al., 2003; Scheffler et al., 2001). These acquisitions are effectively distortion-less, in that they exhibit a similar level of distortion to structural scans. One possibility for 3D imaging is to extend single-line acquisitions to the third k-space dimension (3DFT), but this is relatively slow. Instead, most subsequent work has used 3D readouts that stack k-space planes acquired with either EPI (Chappell et al., 2011; Miller et al., 2006) or spirals (Lee et al., 2007, 2008) in the third k-space dimension.^10^ Although sometimes referred to as “distortion-less”, these scans will exhibit some amount of distortion or blurring, which scales with the duration of the readout (since this determines the amount of phase accrual). One important property of 3D acquisitions is the ability to use parallel imaging to accelerate acquisitions in two dimensions, which enables whole-brain coverage with 1–3-second temporal resolution (Chappell et al., 2011).

One issue with segmented k-space acquisitions in general, and with 3D trajectories in particular, is sensitivity to physiological noise. In cortical areas, pass-band SSFP has been shown to be less sensitive to physiological noise than GRE (Miller et al., 2007). However, my group subsequently discovered that this difference did not hold in areas like the brainstem, and that pass-band SSFP was actually extremely sensitive to physiological noise in these areas when heavily-segmented 3D acquisitions were used. This effect has been demonstrated to result from cardiac pulsation, which introduces phase instabilities in k-space that cause time-varying aliasing (Tijssen et al., 2011). One method for reducing this instability is to synchronize the order of the acquired k-space segments in terms of the cardiac cycle (i.e., in real time), which can reduce the instabilities by up to 40% (Tijssen et al., 2011). However, these methods are inevitably imperfect, and it some residual aliasing may well remain (or other artifacts could be introduced from the reordering itself).

## The real world

To date, the applications of SSFP FMRI have been fairly limited, to the extent that I am not aware of any SSFP FMRI studies that have resulted in new neuroscience findings (as opposed to a demonstration of the sequence's capabilities). In this section I will discuss why this might be, and what will be required of SSFP FMRI to make an impact in neuroscience.

Why has SSFP yet to find a niche in FMRI? In part, this is because other technologies have been able to tackle some of the shortcomings of conventional FMRI that SSFP was proposed to address. Above, I described the primary benefit of SSFP FMRI as the ability to uncouple k-space acquisition from the source of functional contrast, which should enable high-resolution, low-distortion FMRI. However, one could argue that parallel imaging also achieves this in a more limited way by (partially) uncoupling the reconstructed matrix size from the number of acquired data points. Similarly, areas of signal dropout in GRE translate into banding in SSFP, which can be overcome through combination of multiple data sets with different phase cycling. Advanced shimming methods (such as z-shim or real-time shimming) also have potential to mitigate these problems, although again one might argue that there is a limit to what might be achieved. These alternate approaches work in the context of GRE, and do not sacrifice functional contrast or coverage, which is appealing for many applications.

However, I would consider it premature to paint too negative a picture of the future prospects for SSFP FMRI. Recent years have seen several groups investigating the possible benefits of SSFP for specific imaging needs. One compelling application being explored is FMRI of the retinal layers, requiring both high resolution and low distortion. A recent study demonstrated layer-specific BOLD responses in the mouse retina to hypoxic challenges at 45 × 45 × 500 μm resolution (Muir and Duong, 2011). Another preliminary report showed activity in the olfactory bulb, a small structure in a high-susceptibility region of the brain (Parrish et al., 2008). Our group has considered the use of pass-band SSFP the brainstem (Tijssen et al., 2011), where physiological noise poses a particular problem to multi-shot acquisitions. We have found the low levels of pass-band contrast at 3T to be limiting, but a recent study demonstrated robust high-resolution, pass-band SSFP FMRI at 9.4T (Park et al., 2011). In light of these, admittedly limited, demonstrations of the technique's potential, what are the primary challenges facing SSFP FMRI?

For pass-band SSFP, some combination of parallel imaging (Chappell et al., 2011), optimized shimming (Lee et al., 2009) and alternating phase cycling (Patterson et al., 2011) should enable whole-brain coverage in reasonable volume scan times. There is still considerable development to be done in this area. The more troubling issue, at least at low field, is reduced contrast compared to long-TE GRE. It is worth noting that the reduced physiological noise observed in cortical regions at short TR should partially mitigate the reduced BOLD contrast (Miller et al., 2007), but this does not appear to be sufficient to bring contrast-to-noise ratios up to the level of GRE (Zhong et al., 2007). High-field imaging may overcome the limitations of pass-band contrast (Park et al., 2011), but will generally include T2 and T2* contributions unless TR is very short (Miller and Jezzard, 2008; Zhong et al., 2007). At short TR, pass-band SSFP FMRI is dominated by T2 contrast (Miller and Jezzard, 2008), much like spin-echo FMRI. The well-established ability to detect activation with spin echo at high field (Norris, 2012) may bode well for the viability of SSFP, with the benefit over spin echo being the compatibility with reduced readout duration for low distortion.

Transition-band SSFP, on the other hand, remains limited primarily by spatial coverage and sensitivity to physiological noise. Several methods for reducing physiological noise sensitivity have been suggested (Lee et al., 2006a; Tijssen et al., 2011). As with pass-band SSFP, the limited coverage is related to field homogeneity. With shim coils continually evolving to higher orders and the steady advance in parallel transmit technology, transition-band techniques may be more viable in the future. The large signal changes suggest that transition-band SSFP may be particularly useful at low field for localized, high-resolution scanning.

Finally, the techniques discussed here probably only represent a fraction of what is possible for functional imaging with SSFP. There may be some mileage to be gained by considering methods for re-shaping the SSFP profile, for example, to broaden the range of frequency sensitivity (Bieri et al., 2009). It may also be possible to tune the sequence's sensitivity by selectively combining different signal formation pathways (Barth et al., 2008; Park et al., 2011). Given the fascinating range of SSFP techniques that the MRI community has produced over the last 10 years, in and outside the brain, it would be disappointing if new SSFP FMRI techniques did not continue to be proposed in years to come.

## Footnotes

1 Purists may object to my use of SSFP throughout this paper to refer to the balanced technique. However, I find “BSSFP” (or “bSSFP”) visually and verbally jarring, and “balanced SSFP” wordy. I will take the prerogative of the invited paper and use the term coined by Carr in 1958.

2 During the early 2000s, I was fortunate to be a student at Stanford's MRSRL, one of the labs exploring the esoteric and curious properties of SSFP. While most of the lab was interested in cardiac or musculoskeletal imaging, I was singularly focused on the brain, and John Pauly was kind enough to let me take on SSFP for neuroimaging as my PhD topic (largely focused on diffusion imaging). My personal perspective on SSFP FMRI has been inevitably shaped by his lab, which has been a major player, but by no means the only contributor, to these techniques. My somewhat naïve approach to brain imaging was subsequently refined in Oxford's FMRIB Centre, a more familiar name to the *neuro-*imaging community. In the spirit of this Special Issue, I will include the occasional comment about the evolution of these techniques based on my best, occasionally flawed, recollection. Recognizing that many readers may find this chatty and self-indulgent, I will confine these comments to footnotes^2a^. ^2a^Long footnotes (Wallace, 1997).

3 Klaus Scheffler was greatly admired by the graduate students at the MRSRL. Following the lead of Brian Hargreaves and Bill Overall (both doing highly original SSFP work of their own), I made a point to see Klaus speak whenever possible. He had the aura of a magician with SSFP, presenting one clever signal manipulation after another. I have a vague memory of seeing Klaus give a talk on his SSFP FMRI method at the ISMRM, and being very excited. Klaus assures me no such thing ever happened: that he went straight to journal publication and never presented this work at a conference. So much for memory.

4 The year after Klaus Scheffler published his initial idea, I mentioned the work to John Pauly. John is something of an idea factory, and he quickly saw that one could avoid imaging in the low-signal part of the profile by dropping the flip angle and using the phase transition as the source of BOLD contrast. John for once abandoned his tendency for reserved understatement and was direct in encouraging me to pursue the idea. We weren't doing any FMRI at that time, and according to my lab books, I spent a productive 2 weeks setting up stimulus delivery, implementing a new sequence and writing custom GLM analysis code in Matlab. After a few attempts involved misplaced transition bands and a bit of head scratching about magnitude rather than phase changes, we were up and running. A large fraction of my last year at Stanford was spent running and analyzing FMRI experiments with Jongho Lee, then a newly-minted graduate student. Jongho's initial duties involved lying prone in the scanner, but he quickly proved himself more useful outside of the bore. In a few frenzied months, we obtained our first results, applied for a grant and wrote a paper.^4a^ ^4a^In that order, meaning that some of the bravado of my first grant found its way into the paper. Fortunately, Klaus seems not to have taken lasting offense to my over-enthusiasm for promulgating the virtues of our modification of his method.

5 In early 2003, as we were starting to make headway with transition-band FMRI, Graham Wright visited John Pauly's group at Stanford. The three of us spent an hour or so discussing both projects. I don't know whether it was John or Graham who first saw the link, but I must admit I didn't really get it. I suspect this may in part have been because Graham's goal of estimating T2 in whole blood sounded suspiciously like *cardiac* MRI, whereas I was fervently a *brain* person. I do remember being skeptical there would be sufficient contrast in gray matter, particularly at 1.5 T, where we were working. This was little more than a hunch, and it proved to be wrong.

6 In retrospect, it seems a bit dense that I didn't make the connection between Chris’ abstract and Rohan's work, given that I had previously discussed exactly this connection with Graham. Perhaps I was blinded by the different contrast mechanism Chris was proposing. All I can say in my defense is that in an email exchange leading up to the conference, John (who chaired Chris’ oral session) didn't pick up on this, either. So (for a brief period anyway) I was no denser than John Pauly. I can live with that.

7 It is at this point that we encounter the “Two-Lee Problem”, in which two different researchers, Jongho Lee and Jin Hyung Lee, were both publishing SSFP FMRI work in John Pauly's group. They are differentiated in the bibliography as “J. Lee” (Jongho) and “J.H. Lee” (Jin Hyung).

8 A good example of the benefits that can come from changing institution relates to my subsequent years in Oxford working with Peter Jezzard. I was initially disinclined to delve much into BOLD physiology, preferring to focus on the kind of technical developments that Stanford excels at. To Peter, however, the technique couldn't move forward without better understanding of signal mechanisms. It took patient persistence on his part to convince me, but the resulting model evolved into a tremendously satisfying project and is one of my proudest contributions to this area (Miller and Jezzard, 2008).

9 One possibility Steve Smith and I briefly explored is to modulate regressor amplitudes based on a crude, voxel-wise estimate of the variation in functional contrast over time. For example, by estimating the field map drift over time, one could predict a voxel-wise time course of contrast gain. More pressing topics prevailed, but I see no reason why this shouldn't be possible.

10 There are sometimes real and important differences between EPI and spiral readouts, and some work has suggested that SSFP FMRI is better suited to one or the other. Having worked with both, my experience is that either segmented EPI or interleaved spirals can be used with high efficiency, and that by far the most important detail is the quality of implementation on the scanner platform being used.
